# Supplementary figures and images for: Autologous skeletal myoblast patch implantation prevents the deterioration of myocardial ischemia and right heart dysfunction in a pressure-overloaded right heart porcine model
Source: PLoS One. 2021 Feb 26;16(2):e0247381. doi: 10.1371/journal.pone.0247381 (PMC7909703; doi:10.1371/journal.pone.0247381)

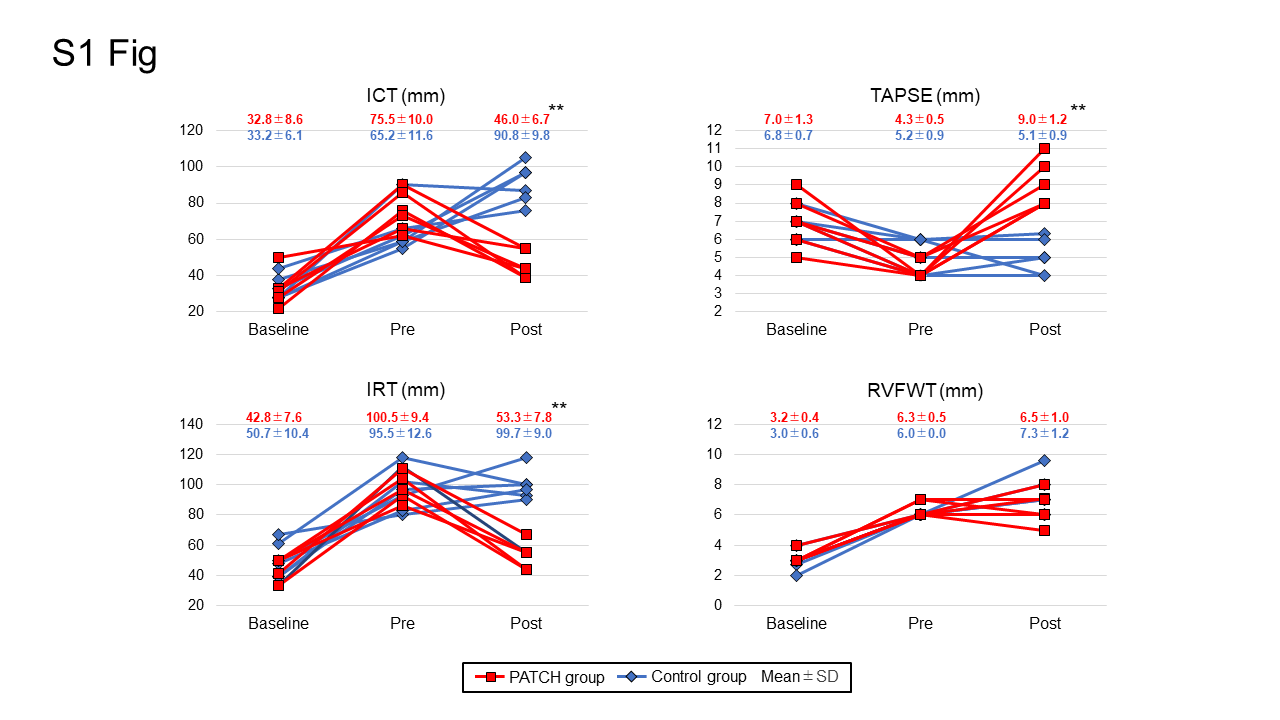

Supplement: S1 Fig — RV systolic and diastolic functions were significantly ameliorated in the PATCH group than in the control group, at two months after patch implantation or sham operation. The red and blue numbers at each point represent the means ± standard deviation of the PATCH group and control group, respectively. P-values were calculated using the Mann-Whitney U test. P<0.01** versus control group. ICT, isovolumic contraction time; TAPSE, tricuspid annular plane systolic excursion; IRT, isovolumic relaxation time; RVFWT, right ventricular free wall thickness. (TIF) [file pone.0247381.s001.tif]

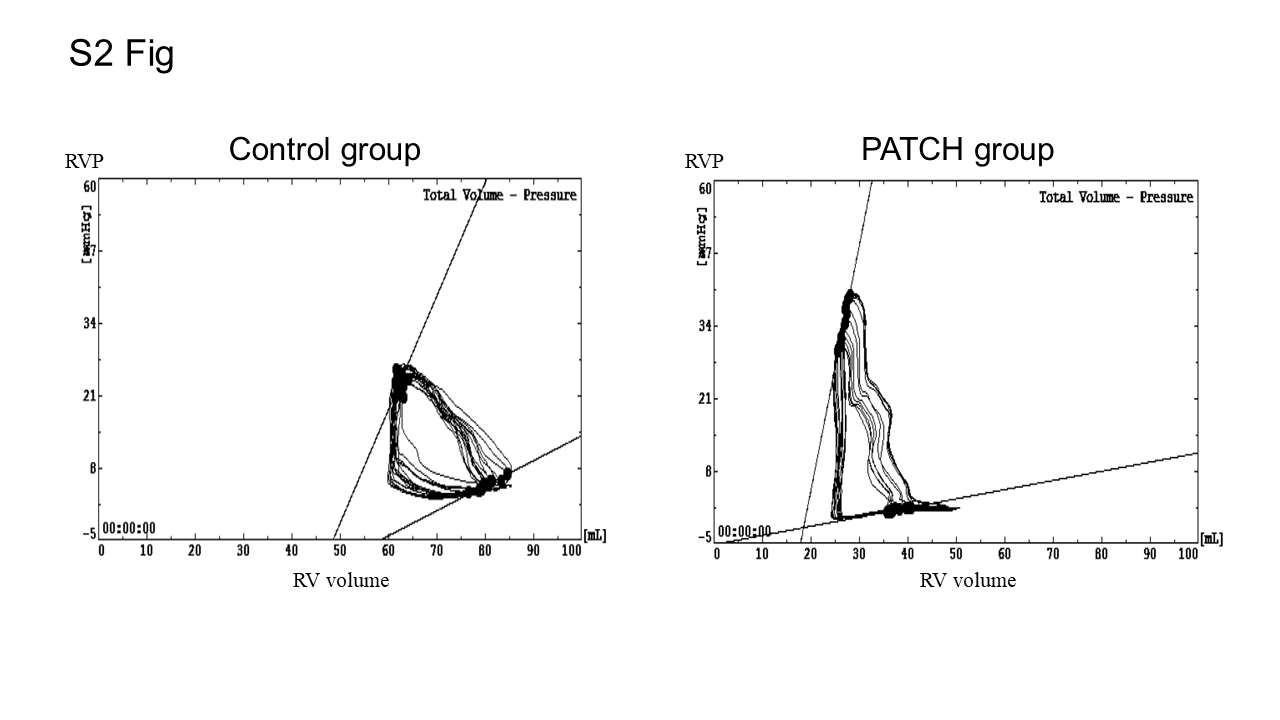

Supplement: S2 Fig — The slope of the end-systolic pressure–volume relationship is displayed as a black straight line on top of the pressure-volume loops. The correlation of the end-diastolic pressure–volume relationship is displayed as a back straight line below the pressure-volume loops. RVP, right ventricular pressure; RV, right ventricle. (TIF) [file pone.0247381.s002.tif]

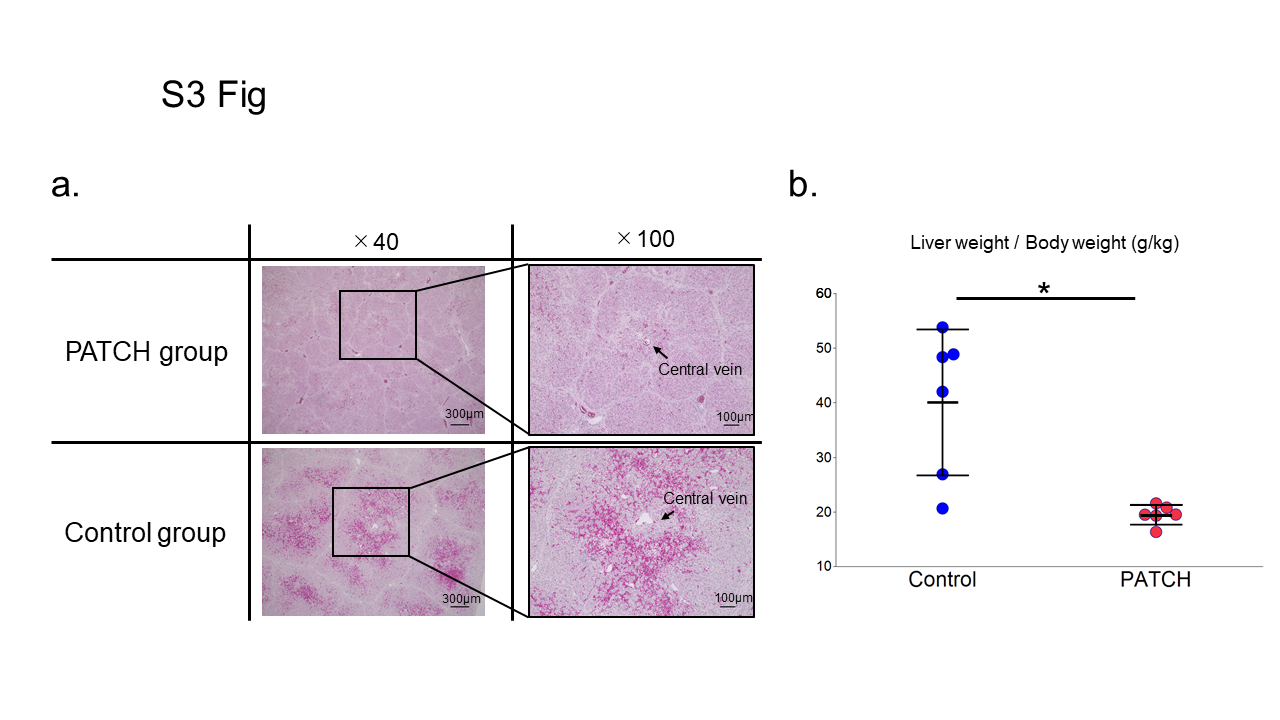

Supplement: S3 Fig — (a) Representative photomicrographs of hematoxylin-eosin staining (×40, scale bar = 300 mm; ×100, scale bar = 100 mm). Congestion around the central vein in the hepatic lobule was more marked in the control group than in the PATCH group. (b) The ratio between the liver mass and the bodyweight of minipigs was significantly lower in the PATCH group than in the control group. The horizontal line in the middle indicates the mean, and the whiskers mark indicates the standard deviation. P-values were calculated using the Mann-Whitney U test. P<0.05*. (TIF) [file pone.0247381.s003.tif]
